# Resistance Affects the Field Performance of Insecticides Used for Control of *Choristoneura rosaceana* in Michigan Apples and Cherries

**DOI:** 10.3390/insects12090846

**Published:** 2021-09-21

**Authors:** Abdulwahab M. Hafez, David Mota-Sanchez, Christine Vandervoort, John C. Wise

**Affiliations:** 1 Pesticides and Environmental Toxicology Laboratory, Plant Protection Department, College of Food and Agriculture Sciences, King Saud University, P.O. Box 2460, Riyadh 11451, Saudi Arabia; 2 Department of Entomology, Michigan State University, 243 Natural Science, East Lansing, MI 48824, USA; motasanc@msu.edu (D.M.-S.); wisejohn@msu.edu (J.C.W.); 3 Pesticide Analytical Laboratory, Michigan State University, 206 Center for Integrated Plant Systems, East Lansing, MI 48824, USA; vanderv2@msu.edu

**Keywords:** obliquebanded leafroller, field-based residual bioassay, pesticide residue analysis, integrated pest management, cherry, apple

## Abstract

**Simple Summary:**

In the years that *Choristoneura rosaceana* was first viewed as a primary pest in fruit orchards, it was routinely targeted with insecticides within integrated pest management (IPM) programs. However, the development of resistance against a number of insecticides in *C. rosaceana* field populations has limited the efficacy of these control programs. One critical component in *C. rosaceana* management is to test whether the detected resistance levels resulted in a practical resistance, i.e., a “lack of control under field conditions” or not. Therefore, this study aimed to identify the field performance in apple and cherry orchards of different insecticides against resistant *C. rosaceana* field populations using field-based residual bioassays and residue analysis. Compounds demonstrating low levels of field-evolved resistance in *C. rosaceana* populations from apple and cherry orchards did not result in practical resistance in the field-based trial (i.e., lack of control under field conditions). However, compounds with high levels of resistance of *C. rosaceana* resulted in practical resistance in both resistant populations. Only chlorantraniliprole and indoxacarb showed long-lasting residues with measurable leaf residues over all post-application intervals while the leaf residues of the other compounds had largely degraded within the first 7 days. These findings can help fruit growers make adjustments to spray/re-application intervals and optimally utilize important chemical tools in their integrated pest management programs.

**Abstract:**

Field-based residual bioassays and residue analysis were conducted to assess the field performance and toxicity longevity of different insecticides that had previously been associated with resistance of *Choristoneura rosaceana* populations collected from apple and cherry orchards. In this study, 12–24 h-old larvae of apple and cherry populations were exposed to apple and cherry leaf samples, respectively, at post-application intervals and a susceptible population served as a reference of each. In the apple and cherry trials, the order of residual longevity of insecticides that effectively controlled the tested populations was as follows: bifenthrin and spinetoram (apple: 14, cherry 21-day post-application), phosmet (apple: 7, cherry 14-day post-application), chlorantraniliprole (apple: 7-day post-application), and indoxacarb and emamectin benzoate (apple: 1, cherry 7-day post-application). Compared to the susceptible population, the resistant populations resulted in a measurable loss of field performance, or “practical resistance”, for the insecticides emamectin benzoate (at 7-day post-application), chlorantraniliprole (at 21-day post-application), and indoxacarb (at all post-application intervals) in the apple trials, while in cherry trial just indoxacarb at 7-day post-application showed a reduced efficacy. In terms of long-lasting residues, only chlorantraniliprole and indoxacarb maintained measurable leaf residues over all post-application intervals while the leaf residues of the other compounds had largely degraded within the first 7 days. These findings can help fruit growers make adjustments to their spray/re-application intervals and optimally utilize important chemical tools in their integrated pest management programs.

## 1. Introduction

The obliquebanded leafroller, *Choristoneura rosaceana* (Harris), is an economical pest of apple and cherry [1]. Even though *C. rosaceana* has a wide host range, with over 50 species [2], for a long time it was considered as a minor pest with limited damage in fruit orchards [3]. However, this perception changed in the late 1970s when outbreaks of *C. rosaceana* populations occurred and it became a serious pest causing economic damages in fruit orchards [4].

In the years that *C. rosaceana* was first viewed as a primary pest in fruit orchards, it was routinely targeted with insecticides within integrated pest management (IPM) programs. The control programs mainly relied on conventional insecticides, especially organophosphates. Eventually, resistance to the conventional insecticides was documented in *C. rosaceana* populations throughout the North American fruit-producing regions [1,3,5,6].

In the mid-1990s, fruit growers replaced many conventional insecticides that belong to the old chemical classes with new reduced-risk insecticides to combat resistance problems and to fulfil the restriction or prevention on the use of many conventional insecticides that was imposed by the 1996 Food Quality Protection Act (FQPA) [1].

Many of the new insecticides that replaced the older chemistries were highly effective against the *C. rosaceana* populations [1,5,7,8,9,10,11]. However, recent studies have documented low to high resistance levels to some new insecticides in the *C. rosaceana* field populations, even though some of these *C. rosaceana* field populations have not been exposed to these new insecticides before [1,3,4,9,12,13].

While baseline laboratory studies to document field-evolved resistance in *C. rosaceana* from commercial fruit orchards are important, the results are often difficult for growers to interpret because it is missing the field efficacy component, or “practical resistance”; i.e., field-evolved resistance that has practical consequences for pest management [14]. Field-based residual bioassays can provide temporal performance data that help demonstrate to growers how various degrees of resistance are expressed under semi-field conditions [15]. Concurrent residue analysis, in turn, can determine the insecticide longevity under normal field conditions. This temporal dimension of the insecticide field performance can help growers to identify adjustments to spray/re-application intervals in their IPM programs [16,17,18,19].

The performance of an insecticide under laboratory conditions is theoretically expected to be a good indicator of its field performance but in reality it varies [20,21]. Examples of compounds with documented “equal effectiveness” under laboratory and field conditions include methoxyfenozide, lambda-cyhalothrin, and acetamiprid against *Cydia pomonella* (Linnaeus) [15]; methoxyfenozide and tebufenozide against *Paralobesia viteana* (Clemens) [22]; spinosad, imidacloprid, and thiacloprid against *Rhagoletis mendax* Curran [23,24,25]; and bifenthrin, malathion, and spinetoram against *Drosophila suzukii* [20]. However, some studies have documented differences between the insecticides’ efficacy under laboratory and field conditions. For instance, clothianidin and thiacloprid against *Cydia pomonella* (Linnaeus) [15,16], as well as imidacloprid and spinosad against *Rhagoletis pomonella* (Walsh) [26] showed lower effectiveness under field conditions compared to the laboratory conditions. By contrast, thiacloprid against *R. pomonella* showed higher efficacy under field conditions compared to the laboratory conditions [27].

Therefore, this study aimed to:

(1) Identify the performance of different insecticides against resistant *C. rosaceana* field populations originating from apple and cherry using a field-based residual bioassay and compare it with their performance in laboratory-based bioassays.
(2) Assess the toxicity longevity of the different insecticides against *C. rosaceana* field populations in apple and cherry using field-based residual bioassays and residue analysis.

## 2. Materials and Methods

### 2.1. Insects

Three *C. rosaceana* populations were tested in the bioassays. In summer 2013, one *C. rosaceana* field population was collected from a commercial apple and another *C. rosaceana* field population was collected from a commercial cherry orchard located in Kent and Newaygo Counties in western Michigan, respectively. The third population was a laboratory-susceptible population. Following the method of Hafez et al. [1], the three *C. rosaceana* populations were reared and tested under constant conditions (25 ± 1 °C, 70% RT, and photoperiod of 16:8 h light:dark).

### 2.2. Field Residual Activity Trials

**Figure 1:**
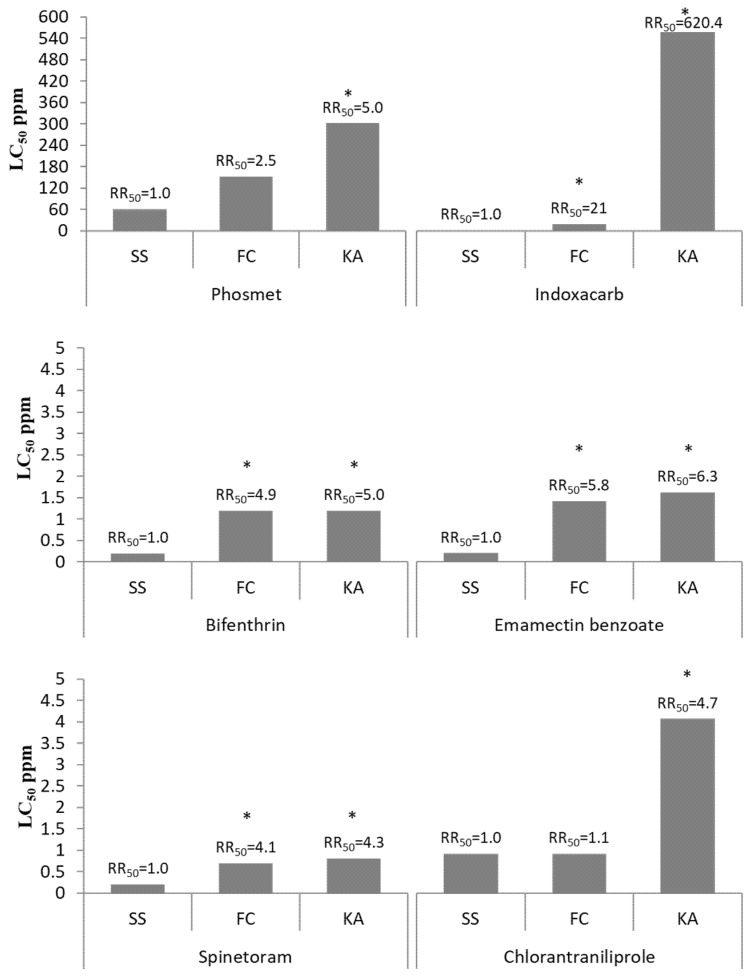
The baseline toxicity study of Hafez et al. [1] of the tested insecticides in the current study on 12–24 h-old *C. rosaceana* larvae of two field strains, namely, apple (KA) and cherry (FC), compared to a susceptible strain (SS). LC_50_ = median lethal concentration. PPM = part per million. Resistance ratio_50_ (RR_50_) = LC_50_ value of the field strain/LC_50_ value of the susceptible strain. An asterisk (*) means the field strain is significantly more resistant than the susceptible strain.

Apple and cherry field trials were conducted on 9 June and 16 July 2015, respectively, at the TNRC in Fennville, Michigan (42°35′42.4″ N, 86°09′22.0″ W). The treatments were selected based on the results of the toxicity baseline study of Hafez et al. [1] (Figure 1), choosing compounds with no to a high level of resistance. In the apple trial, six treatments of insecticides (phosmet, bifenthrin, spinetoram, chlorantraniliprole, indoxacarb, and emamectin benzoate; for full details, see Table 1) plus a control treatment (water only) were applied to 28-year-old semi-dwarf Red Delicious apple trees (Malus Miller; Rosaceae) with a six-meter row spacing and three-meter tree spacing (6 m × 3 m). In the cherry trial, five treatments of insecticides (phosmet, bifenthrin, spinetoram, indoxacarb, and emamectin benzoate; for full details, see Table 1) plus a control treatment (water only) were applied to 21-year old Montmorency cherry trees (Sare Montmorency) with six-meter row spacing and 4.5-m tree spacing (6 m × 4.5 m). We used an airblast sprayer (model 1029, FMC Corp., Jonesboro, Arkansas, USA) operated at 4 km/h with three nozzles on a side, and 935 L/ha (nozzle disc# 4, 3 hole ceramic whirl plate) (Durand-Wayland Inc., LaGrange, GA, USA) and 1242 kPa, to apply the tested insecticides (Table 1). The airblast sprayer was first calibrated to ensure the delivery of 935 L/ha (100 gallons per acre) of water plus test material spray solution.

Five single tree replications were used for each treatment and were arranged in a randomized complete block design (RCBD). In order to prevent insecticide drift between the adjacent treatments, one buffer (untreated) row was used to separate the treatment blocks and two buffer trees were used to separate the treated trees in each block. To obtain a representative sample, each replication was divided into sides representing the four cardinal directions and each side was divided into two levels (upper and lower). At 1, 7, 14, and 21-day post-application, a total of 48 young and fully expanded leaves were collected from each replication (12 from each side/six from each level). Collected samples were placed in tight-sealing plastic bags (the six leaves from each level of each side were collected in a separate bag for a total of eight bags per replicate), placed in coolers, and transferred to the lab to conduct the laboratory assays.

### 2.3. Field-Based Residual Bioassay

Following the method of VanWoerkom, et al. [28], residual toxicity bioassays were conducted as soon as the samples arrived in the lab. One leaf was selected randomly from each bag, each one representing a level and side of each tree, for a total of eight leaves per replicate. Using a cork borer, a 20-mm-diameter disc was cut from each leaf. The cork borer was dipped in acetone between each sample to minimize cross contamination. The eight discs were placed in a 50-mm-diameter Falcon^®^ disposable petri dish (Corning Inc.-Life Science. Durham, NC, USA) padded with moistened (by deionized water) 55-mm-diameter Whatman^TM^ filter paper (Whatman Limited, Buckinghamshire, UK). Five 12–24 h-old *C. rosaceana* larvae were placed in each petri dish. Five petri dishes were assigned to each treatment (one for each replication). The petri dishes were maintained under constant conditions (25 ± 1 °C, 70% RT, and photoperiod of 16:8 h light:dark). The *C. rosaceana* larvae from the apple population were exposed to treated apple leaves, the *C. rosaceana* larvae from the cherry population were exposed to treated cherry leaves, and the *C. rosaceana* larvae from the susceptible population were exposed to the treated apple leaves in the apple trial and to the treated cherry leaves in the cherry trial. The larval mortality was recorded 120 h after the larvae were placed in the petri dishes for all treatments except for the non-neurotoxic insecticide chlorantraniliprole treatment, where it was recorded after 168 h because it has a slower mode of action compared to the neurotoxic insecticides. Any larva failing to move when touched with a soft camel’s hair brush was considered dead.

### 2.4. Residues Analysis

When the samples arrived at the lab, 20 g ± 1 (approximately 40 leaves) were removed from each replication/each treatment (total of three replications for each treatment) for residual analysis. Leaves were stored in 120 mL glass jars (Qorpak, Bridgeville, PA, USA) containing 8.0 g Mg_2_SO_4_ to absorb water in the sample and 2.0 g NaCl to push the ionized compounds into the water. Then HPLC-grade dichloromethane was added until the entire sample was submerged (approximately 50–100 mL) and the jars were stored at 4 °C until the samples were processed.

After 2–4 weeks, the contents of each jar were filtered into a 250 mL round-bottom flask through 185-mm-diameter filter paper containing 20 g of anhydrous sodium sulfate to remove the water from the sample. After completing the filtration, the 250 mL round-bottom flasks were connected to a rotary evaporator (R-114 rotary evaporator, Büchi Labortechnik AG, Flawil, Switzerland) to remove the dichloromethane. In total, 2 mL of HPLC-grade acetonitrile was added to dissolve the remaining dry extract residue and the flask was rotated for 3 min. Using a 3 mL syringe, we collected the dissolved extract residue and then filtered it through a syringe filter (45-mm Acrodisc 33 mm, Pall, East Hills, NY, USA) into 2 mL HPLC glass vial (Agilent Technologies Inc., Santa Clara, CA, USA). The purpose of this filtration was to remove the remaining particulates. The 2 mL HPLC glass vials then were stored at 4 °C until the samples were analyzed. High-performance liquid chromatography (HPLC) was used to analyze the samples for the spinetoram and emamectin benzoate treatments and gas chromatography (GC) to analyze the samples for the phosmet, bifenthrin, chlorantraniliprole, and indoxacarb treatments. The limit of detecting a peak (LOD) and limit of quantifying a peak (LOQ) values for each treatment compound are presented in Table 2.

### 2.5. Statistical Analysis

When needed and prior to the analysis of the field-based residual bioassay data, mortalities were corrected based on the mortality of the control treatment using the formula of Abbott [29]. The mortality data of the field-based residual bioassay results for each insecticide were analyzed separately, and were analyzed by a repeated-measure analysis of variance (ANOVA) using PROC MIXED in SAS [30] and mean separation using an LSD α = 0.05. For each insecticide, the mean percentage mortality of *C. rosaceana* 12–24 h-old larvae of each field population and the susceptible population was compared at each post-application interval and the mean percentage mortality of each population was compared over the post-application intervals. The residue data of each insecticide at different post-application intervals were analyzed by a repeated-measure analysis of variance (ANOVA) using PROC MIXED in SAS [30] and a mean separation using an LSD α = 0.05.

## 3. Results

The main objective of the current study was to determine how resistance affects the field performance of insecticides used for the control of *C. rosaceana* in relation to the associated residual activity of each compound. The data for each compound were analyzed and compared separately for the apple and cherry collected populations.

### 3.1. Apple Trial

#### 3.1.1. Phosmet

**Figure 2:**
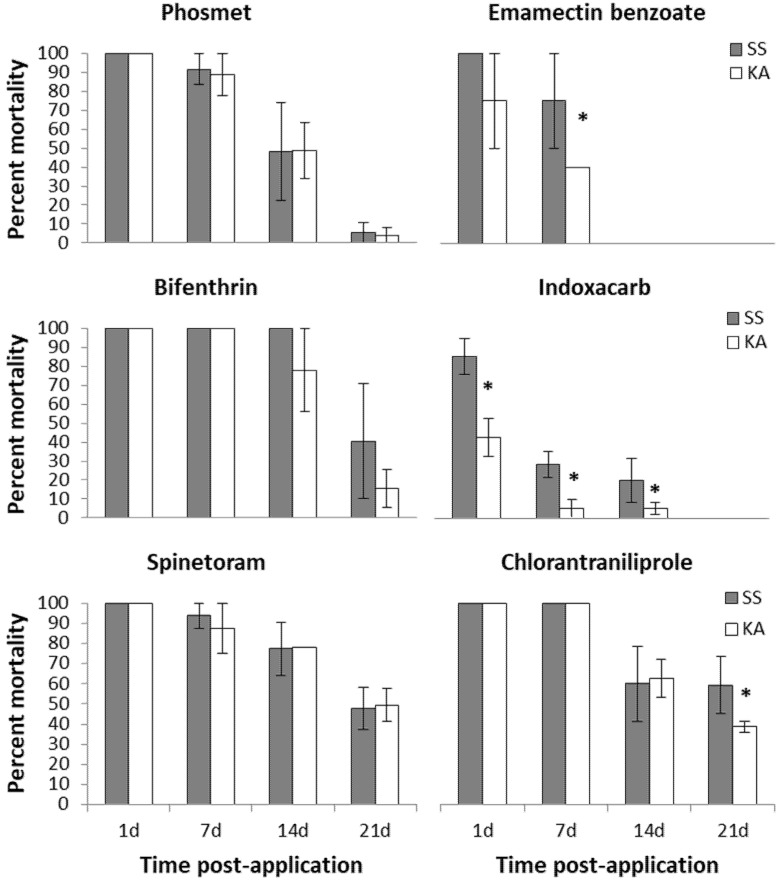
Mortality means (± SE) of the *C. rosaceana* 12–24 h-old larvae of the apple population (KA) and susceptible population (SS) when exposed to apple foliage collected at different post-application intervals. An asterisk (*) means the mortality of the apple population is significantly different from the mortality of the susceptible population at a given post-application interval (α = 0.05).

In the field-aged residue bioassays, no significant differences were found between the larval mortality of susceptible and apple *C. rosaceana* populations when exposed to the phosmet-treated apple leaves at all post-application intervals: 1, 7, 14, and 21-days (Figure 2).

**Figure 3:**
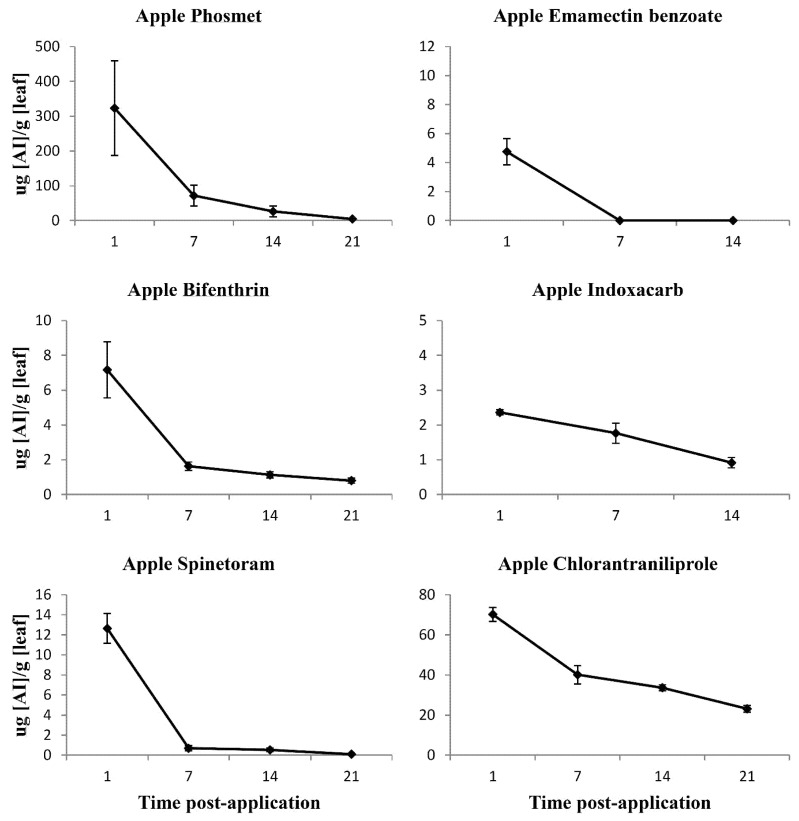
Residue means (SE±) measured in micrograms per gram of active ingredient per leaf taken at 1, 7, 14, and 21-day post-application.

Phosmet toxicity to both the *C. rosaceana* populations were high for the first 7-day post-application, with a loss of performance following closely the gradual decline in surface residues over 21 days (Figure 2 and Figure 3). The efficacy of phosmet declined significantly after 7-day post-application with only 48.3 ± 25.9 and 48.7% ± 14.7 larval mortality in susceptible and apple *C. rosaceana* populations, respectively. By 21-day post-application, its efficacy was diminished completely, with 5.5 ± 5.5 and 4.1% ±4.1 larval mortality in the susceptible and apple *C. rosaceana* populations, respectively (Figure 2).

Gradual degradation was recorded in the phosmet leaf residues over the post-application intervals 1, 7, 14, and 21-day post-application (Figure 3). The major residue degradation occurred within the first 7 days, with only 22.2% of the 1-day post-application leaf residue detected at 7-day post-application (Figure 3).

#### 3.1.2. Bifenthrin

In the field-aged residue bioassays no significant differences were found between the larval mortality of susceptible and apple *C. rosaceana* populations when exposed to the bifenthrin-treated apple leaves at all post-application intervals: 1, 7, 14, and 21 days (Figure 2).

Bifenthrin toxicity to the *C. rosaceana* populations was high for the first 14-day post-application, despite the gradual decline in surface residues (Figure 2 and Figure 3). Its efficacy against both populations required nearly three weeks to show the first significant decline at 21-day post-application, with 40.5 ± 30.4 and 15.6% ±10.0 larval mortality in the susceptible and apple *C. rosaceana* populations, respectively (Figure 2).

Gradual degradation was recorded for the bifenthrin leaves’ surface residues over the post-application intervals 1, 7, 14, and 21-day post-application (Figure 3). The major residue degradation occurred within 7 days, with only 22.8% of the 1-day post-application surface residues detected 7-day post-application (Figure 3).

#### 3.1.3. Spinetoram

In the field-aged residue bioassays no significant differences were found between the larval mortality of susceptible and apple *C. rosaceana* populations when exposed to the spinetoram-treated apple leaves at all post-application intervals: 1, 7, 14, and 21 days (Figure 2).

Spinetoram toxicity to the *C. rosaceana* tested populations was high for 21-day post-application, despite the significant sharp degradation in leaf residues (Figure 2 and Figure 3), with high efficacy against both the *C. rosaceana* tested populations for nearly three weeks post-application. The first significant loss of performance was observed at 21-day post-application with 47.7 ± 10.5 and 49.3% ± 8.2 larval mortality in susceptible and apple *C. rosaceana* populations, respectively (Figure 2).

Sharp degradation was recorded in the spinetoram leaf residues over the post-application intervals 1, 7, 14, and 21 days (Figure 3). Negligible spinetoram residues were detected at 7, 14, and 21-day post-application (Figure 3). The most dramatic residue degradation occurred within the first 7 days, with only 5.5% of the 1-day post-application surface residue detected 7-day post-application (Figure 3).

#### 3.1.4. Chlorantraniliprole

In the field-aged residue bioassays no significant differences were found between the larval mortality of susceptible and apple *C. rosaceana* populations when exposed to the chlorantraniliprole-treated apple leaves at the post-application intervals 1, 7, and 14 days (Figure 2). However, at 21-day post-application, a significant difference in chlorantraniliprole performance was detected between the susceptible and apple *C. rosaceana* populations where the larval mortality was significantly lower for the apple population compared to the susceptible population when both were exposed to the 21-day post-application treated apple leaves (Figure 2).

Chlorantraniliprole toxicity to the *C. rosaceana* tested populations was high for the first 7-day post-application, with a loss of performance following closely the gradual decline in surface residues over 21 days (Figure 2 and Figure 3). Its efficacy against both *C. rosaceana* populations declined significantly after 7-day post-application with only 60 ± 18.7 and 62.6% ± 9.6 larval mortality in susceptible and apple *C. rosaceana* populations, respectively (Figure 2).

Gradual degradation was recorded in the chlorantraniliprole leaf residues over the post-application intervals 1, 7, 14, and 21-day post-application (Figure 3). The major residue degradation occurred within the first 7 days, with only 57.2% of the 1-day post-application surface residue detected 7-day post-application (Figure 3).

#### 3.1.5. Indoxacarb

In the field-aged residue bioassays significant differences in indoxacarb performance were detected between the susceptible and apple *C. rosaceana* populations at all post-application intervals: 1, 7, and 14 days (Figure 2). The larval mortality of the apple *C. rosaceana* population was significantly lower than that of the susceptible *C. rosaceana* population at all post-application intervals (Figure 2).

With the exception of the 85% ± 9.6 larval mortality recorded for susceptible *C. rosaceana* population at 1-day post-application, low toxicity of indoxacarb to the *C. rosaceana* tested populations was recorded at all post-application intervals (Figure 2).

Relatively flat degradation was recorded in the indoxacarb leaf residues over the post-application intervals 1, 7, and 14-day post-application with no major residue declines at any post-application interval (Figure 3).

#### 3.1.6. Emamectin Benzoate

In the field-aged residue bioassays no significant differences were found between the larval mortality of susceptible and apple *C. rosaceana* populations when exposed to the emamectin benzoate-treated apple leaves at 1-day post-application (Figure 2). However, at 7-day post-application, a significant difference in emamectin benzoate performance was detected between the susceptible and apple *C. rosaceana* populations, where the larval mortality was significantly lower for the apple population compared to the susceptible population (Figure 2). No larval mortality was observed in the susceptible and apple *C. rosaceana* populations when both were exposed to the 14-day post-application treated apple leaves (Figure 2).

Emamectin benzoate toxicity was high 1-day post-application to susceptible and apple *C. rosaceana* populations and for the first 7 days to susceptible *C. rosaceana* population (Figure 2), with a loss of performance following closely the rapid decline in surface residues (Figure 2 and Figure 3). Its efficacy against the susceptible laboratory *C. rosaceana* population declined significantly after 7-day post-application, with 0.0% ± 0.0 larval mortality (Figure 2), while efficacy against the apple population declined significantly after 1-day post-application, with 40.0% ± 0.0 larval mortality (Figure 2).

Emamectin benzoate showed a rapid decline in surface residues under field conditions, with leaf residues detected only at 1-day post-application and no leaves surface residues detected at 7 and 14-day post-application (Figure 3).

### 3.2. Cherry Trial

#### 3.2.1. Phosmet

**Figure 4:**
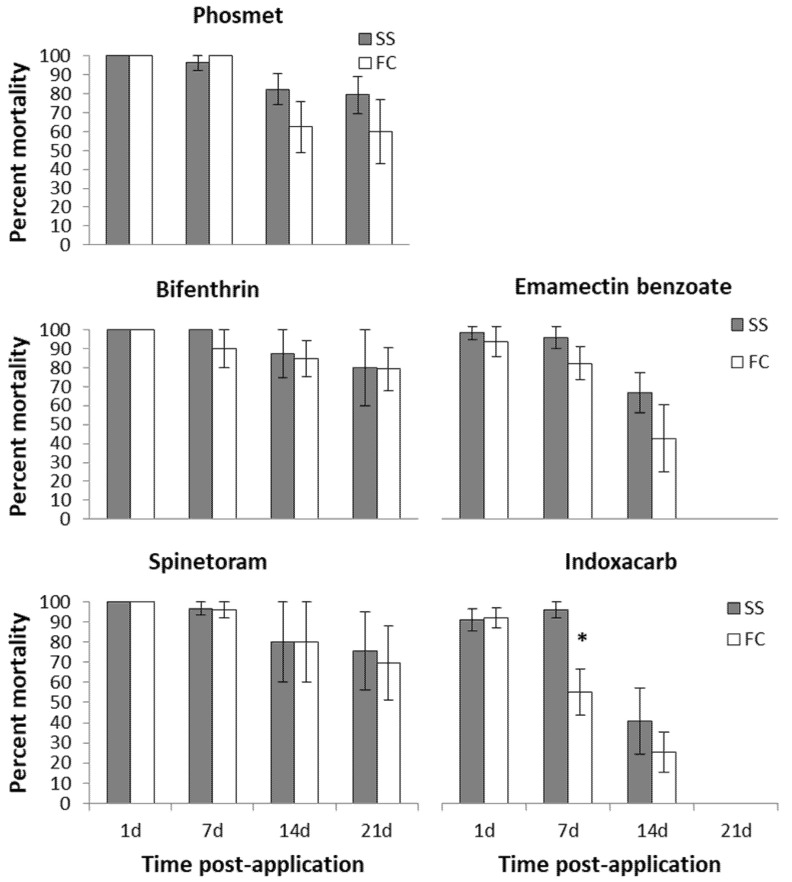
Mortality means (± SE) of the *C. rosaceana* 12–24 h-old larvae of the cherry population (FC) and susceptible population (SS) when exposed to cherry foliage collected at different post application intervals. An asterisk (*) means the mortality of the cherry population is significantly different from the mortality of the susceptible population at a given post-application interval (α = 0.05).

**Figure 5:**
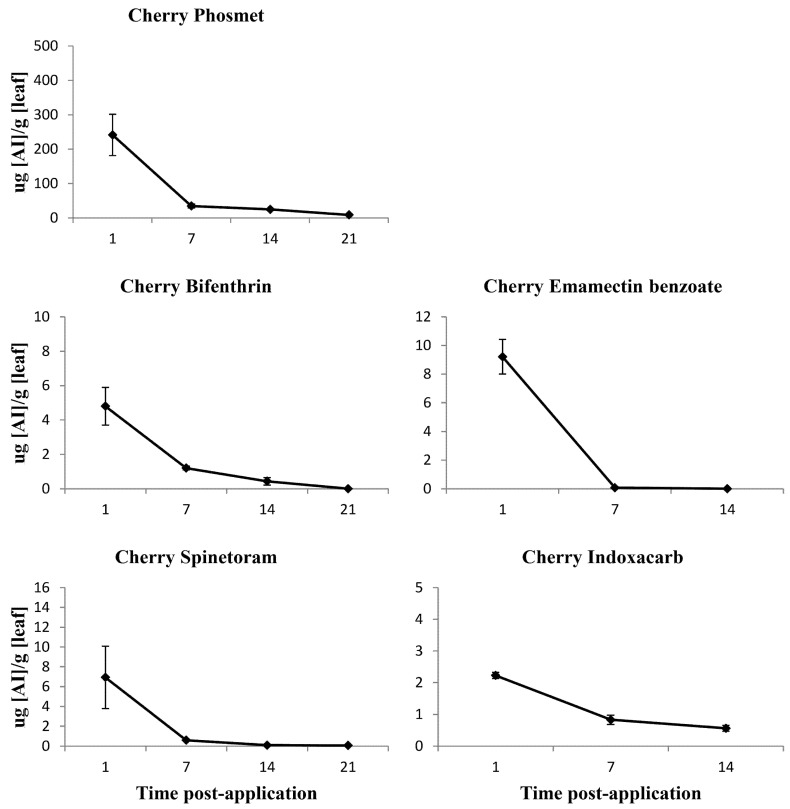
Residues means (SE±) measured in micrograms per gram of the active ingredient per leaf taken at 1, 7, 14, and 21-day post-application.

In the field-aged residue bioassays no significant differences were found between the larval mortality of susceptible and cherry *C. rosaceana* populations when exposed to the phosmet-treated cherry leaves at all post-application intervals: 1, 7, 14, and 21 days (Figure 4). Phosmet toxicity to *C. rosaceana* populations were high for the first 7-day post-application, with a loss of performance following closely the gradual decline in leaf residues over 21 days (Figure 4 and Figure 5). Its efficacy against both *C. rosaceana* populations declined significantly after 7-day post-application, with only 82.2 ± 8.0 and 62.2% ± 13.6 larval mortality in susceptible and cherry *C. rosaceana* populations, respectively (Figure 4). No further significant loss of performance was recorded beyond 7 days. The larval mortality of both *C. rosaceana* populations at 21-day post-application had not declined significantly compared to their larval mortality at 14-day post-application (Figure 4).

Gradual degradation was recorded in the phosmet leaf residues over the post-application intervals 1, 7, 14, and 21 days (Figure 5). The major residue degradation occurred within the first 7 days, with only 14.4% of the 1-day post-application residue detected at 7-day post-application (Figure 5).

#### 3.2.2. Bifenthrin

In the field-aged residue bioassays no significant differences were found between the larval mortality of susceptible and cherry *C. rosaceana* populations when exposed to the bifenthrin-treated cherry leaves at all post-application intervals: 1, 7, 14, and 21 days (Figure 4).

Bifenthrin toxicity to *C. rosaceana* cherry and susceptible populations was high for 21-day post-application, despite the gradual decline in leaf residues (Figure 4 and Figure 5). Its efficacy against both the *C. rosaceana* tested populations did not significantly decline even after 21-day post-application, with 80.0 ± 20 and 79.2% ± 11.4 larval mortality in the susceptible and cherry *C. rosaceana* populations, respectively (Figure 4).

Significant gradual degradation was recorded in the bifenthrin leaf residues from Days 1 to 21 post-application (Figure 5). The major residue degradation occurred within the 7-day post-application, with only 25% of the 1-day post-application residue detected at 7-day post-application (Figure 5).

#### 3.2.3. Spinetoram

In the field-aged residue bioassays no significant differences were found between the larval mortality of susceptible and cherry *C. rosaceana* populations when exposed to the spinetoram-treated cherry leaves at all post-application intervals: 1, 7, 14, and 21 days (Figure 4).

Spinetoram toxicity to *C. rosaceana* cherry and susceptible populations was high 21-day post-application, despite the sharp degradation of the residues (Figure 4 and Figure 5), with a high efficacy against both the *C. rosaceana* tested populations for nearly three weeks’ post-application. The first significant loss in performance was observed at 21-day post-application with 75.7 ± 19.4 and 69.6% ± 18.3 larval mortality in the susceptible and cherry *C. rosaceana* populations, respectively (Figure 4).

Significant, sharp degradation was recorded in the spinetoram leaf residues over the post-application intervals 1, 7, 14, and 21 days (Figure 5). Negligible spinetoram leaf surface residues were detected at 7, 14, and 21-day post-application (Figure 5). The major residue degradation occurred within the 7-day post-application in which only 8.6% of the 1-day post-application residue was detected at 7-day post-application (Figure 5).

#### 3.2.4. Indoxacarb

In the field-aged residue bioassays significant differences in indoxacarb performance were detected between the susceptible and cherry *C. rosaceana* populations at 7-day post-application (Figure 4). The larval mortality of the cherry *C. rosaceana* population was significantly lower than the larval mortality of the susceptible *C. rosaceana* population when both were exposed to the indoxacarb-treated cherry leaves at 7-day post-application (Figure 4). No significant differences were found between the larval mortality of susceptible and cherry *C. rosaceana* populations when they were exposed to the indoxacarb-treated cherry leaves at 1 and 14-day post-application (Figure 4).

Indoxacarb toxicity was high to the susceptible and cherry *C. rosaceana* populations at 1 and 7-day post-application, despite the relatively flat degradation of the residues (Figure 4 and Figure 5). Its efficacy against the susceptible *C. rosaceana* population declined significantly after 7-day post-application with 40.9% ± 16.3 larval mortality (Figure 4), while efficacy against the cherry *C. rosaceana* population declined significantly after 1-day post-application, with a 55.3% ± 11.4 larval mortality (Figure 4).

Slow degradation was recorded in the indoxacarb leaf residues over the post-application intervals 1, 7, and 14-day post-application, with no major residue degradation at any post-application interval (Figure 5).

#### 3.2.5. Emamectin Benzoate

In the field-aged residue bioassays no significant differences were found between the larval mortality of susceptible and cherry *C. rosaceana* populations when exposed to the emamectin benzoate-treated cherry leaves at all post-application intervals: 1, 7, and 14-days (Figure 4).

Emamectin benzoate toxicity to the cherry and susceptible *C. rosaceana* populations was high for the first 7-day post-application, with a loss of performance following closely the rapid decline in residues over 21 days (Figure 4 and Figure 5). Its efficacy against both the *C. rosaceana* populations declined significantly after 7-day post-application, with only 65.4 ± 10.4 and 41.9% ± 17.5 larval mortality in the susceptible and cherry *C. rosaceana* populations, respectively (Figure 4).

Emamectin benzoate showed a rapid decline in residues in leaves under the field conditions, with residues detected at 1-day post-application, but no residues detected at 7 and 14-day post-application (Figure 5).

## 4. Discussion

The current study demonstrated how various degrees of laboratory-documented resistance are expressed under semi-field conditions. The results for the compounds in this study fall into two categories in terms of how resistance is expressed under field conditions: (1) reduced longevity of control; and (2) no evidence for loss of lethality of the compound. For example, phosmet, spinetoram, and bifenthrin were associated with significant levels of resistance in the *C. rosaceana* field populations in the baseline laboratory bioassay (Figure 1) but the field-based residual bioassay revealed that these laboratory-documented significant levels of resistance did not result in a measurable loss of field performance, or practical resistance, with the labeled field rate of those compounds. While the principles of resistance management would justify efforts to rotate to insecticides with different modes of action, our study suggests that the low-level resistance of *C. rosaceana* may not be readily recognized for a normally expected control period of 14 days under grower field conditions.

The differences in the field performance of the same compound under apple and cherry field trials are expected to be due to one or more of the following three factors. First, the tested apple and cherry *C. rosaceana* field populations showed different levels of resistance against the tested insecticides under the baseline study (Figure 1). Second, both field trials were conducted at different times during the same season with slightly different weather conditions, which could impact residues. For example, the apple field trial received 0.0, 2.4, 4.6, and 4.9 milliliters of precipitation over 1, 7, 14, and 21-day post-application, respectively, while the cherry field trial received 0.1, 0.7, 0.7, and 1.6 milliliters of precipitation over 1, 7, 14, and 21-day post-application, respectively. Third, differences in physiology or morphology of the apple and cherry leaves may affect, for example, larvae consumption, insecticide solution adhesion, insecticide penetration, etc. [31].

In addition, the residue sample preparation methods did not grind the leaf substrate within the solvent; thus, depending on the compound, some portions of sub-cuticular residues from the internal leaf tissues may have remained unmeasured. This can likely explain the cases where temporal lethality continued beyond the dates of detectible residues, such as the phosmet, bifenthrin, and spinetoram treatments.

### 4.1. Phosmet

This organophosphate insecticide is a broad-spectrum and effective compound in fruit orchards, including apple and tart cherry, to a wide range of insect pests. Phosmet is slightly soluble in water, has a relatively low vapor pressure, and is stable to photolysis [32,33]. Despite its slight solubility in water, phosmet is highly susceptible to wash-off from precipitation [34,35]. However, its capability of plant cuticle penetration helps to reserve a portion of the phosmet active ingredient in the plant leaves and fruit cuticle [36].

The ability of this wash-off-susceptible compound to maintain longevity over 14-day post-application is possibly due to its nature as a fast-acting contact neurotoxin, where the lethal effect is accumulated through the mobility of the larvae over the treated surface [34] and possibly due to its capability of cuticle penetration [36].

The absence of significant differences between the larval mortality of susceptible and apple *C. rosaceana* populations when exposed to the phosmet-treated apple leaves at all post-application intervals indicate that the documented 5-fold resistance in the field *C. rosaceana* apple population against phosmet in the baseline study (Figure 1) may not result in a measurable loss of its field performance at the labeled field rate.

A similar field performance of phosmet and some other organophosphate insecticides has been documented in previous studies. For example, azinphos-methyl and phosmet effectively controlled first-instar oriental fruit moth larvae for 14-day post-application when they were exposed to treated peach foliage [37]; phosmet effectively controlled apple maggot larvae when they were exposed to treated apple fruit [38]; and azinphos-methyl effectively controlled plum curculio adults when they were exposed to treated tart cherry fruit [39].

In contrast, a different field performance of phosmet and some other organophosphate insecticides has also been documented in previous studies. For example, a high field performance of azinphos-methyl and phosmet was recorded in a tart cherry field study, where both controlled plum curculio larvae for more than 30-day post-application when the larvae were exposed to treated cherry fruit [40]. Chlorpyrifos and azinphos-methyl in two separate apple field studies showed a low field performance, where both failed to control *C. rosaceana* and codling moth neonate larvae for 10 and 14-day post-application, respectively [6,15]. Similarly, malathion showed low field performance by controlling *Drosophila suzukii* for only the first 7-day post-application when the flies were exposed to treated blueberry and strawberry fruit [20].

The current study showed phosmet as an effective chemical tool to control *C. rosaceana* field populations in Michigan apple and cherry orchards. However, this compound should be avoided for controlling *C. rosaceana*, especially with its long history of use in Michigan apple and cherry orchards, due to the high potential of rapid build-up of resistance. If circumstances necessitate applying phosmet, a 7-day post-application should be considered for the re-application interval for this compound.

### 4.2. Bifenthrin

Bifenthrin is a non-systemic, broad-spectrum, and widely used insecticide. The high field-performance of bifenthrin, under the current study, is compatible with its physical properties of being relatively insoluble in water, stable to hydrolysis, and with minimal volatility [41]. Bifenthrin is also moderately susceptible to wash-off from precipitation [34] but its capability of plant cuticle penetration helps to reserve a portion of its active ingredient in the plant leaves and fruit cuticle [36], which may provide an additional support to its physical properties to show this high field performance.

The absence of significant differences between the larval mortality of susceptible and field *C. rosaceana* populations in the apple and cherry trials when exposed to the bifenthrin-treated apple and cherry leaves at all post-application intervals indicate that the documented 5- and 4.9-fold resistance in the field *C. rosaceana* apple and cherry populations, respectively, against bifenthrin in the baseline study (Figure 1) may not result in a measurable loss in field performance at the labeled field rate.

Variable field performance of bifenthrin and some other pyrethroid insecticides have been documented in previous studies. For example, in berry crop field studies, bifenthrin and fenpropathrin showed poor field performance when both failed completely to control *Drosophila suzukii* at 7-day post-application when the flies were exposed to treated blueberry and strawberry fruit [20]. Similarly, esfenvalerate showed a poor field performance when it failed to control *C. rosaceana* at 10-day post-application when the neonate larvae were exposed to treated apple foliage [6]. Permethrin, in turn, showed a moderate field performance by effectively controlling the oriental fruit moth over 14-day post-application when the first-instar larvae were exposed to treated peach foliage [37]. Lambda-cyhalothrin also showed a moderate field performance by effectively controlling codling moth until its efficacy significantly declined when the neonate larvae were exposed to 21-day post-application treated apple fruit [15].

The variation in the field performance of bifenthrin and some other pyrethroid insecticides documented previously and in the current study, is assumed to be due to the differences in the field and application conditions, tested species, tested life-stage, levels of resistance, and plant substrate.

The high field performance of bifenthrin that was documented in the current study indicates that this insecticide is an effective chemical tool to control *C. rosaceana* field populations in Michigan apple and cherry orchards. However, this compound belongs to a chemical class that has a long history of use in Michigan apple and cherry orchards. Therefore, bifenthrin should be avoided for controlling *C. rosaceana* due to the high potential of rapid build-up of resistance. If circumstances necessitate applying bifenthrin, a 14-day post-application should be considered for the re-application interval for this compound.

### 4.3. Spinetoram

Spinetoram is a broad-spectrum and widely used insecticide. It has low solubility in water and a low vapor pressure, but this semi-synthetic compound, isolated from fermentation of *Saccharopolyspora spinose*, degrades rapidly by photolysis [42]. Despite the photolysis sensitivity, spinetoram has excellent translaminar movement where this insecticide has the ability to penetrate the leaf cuticle, moving into and across leaf tissues. This means the compound’s active ingredient is protected from photolysis inside the leaf tissue and this is probably the main reason for the long-lasting control of this compound under field conditions.

The absence of significant differences between the larval mortality of susceptible and field *C. rosaceana* populations in the apple and cherry trials when exposed to the spinetoram-treated apple and cherry leaves at all post-application intervals indicate that the documented 4.3- and 4.1-fold resistance in the field *C. rosaceana* apple and cherry populations, respectively, against spinetoram in the baseline study (Figure 1) may not result in a measurable loss in its field performance at the labeled field rate.

Similar to the current study, the high field performance of spinetoram and spinosad has been documented in previous studies. For example, spinosad showed high field performance with high levels of mortality through 21-day post application in *C. rosaceana*, *P. pyrusana*, *L. subjuncta*, and *C. pomonella* when the larvae were exposed to treated apple fruit and leaves [16]. Spinosad bait as well showed a high field performance when the 14-day post-application treated cherry leaves killed 100% of *Rhagoletis indifferens* adults [26]. A higher spinetoram field performance, compared to its field performance in the current study, was documented by SialandBrunner [7], who reported that the spinetoram-treated apple foliage caused 100% mortality in the *C. rosaceana* neonate larvae (˂48 h old) over 59-day post-application. Since both studies had tested the same compound (spinetoram), species (*C. rosaceana*), life-stage (˂48 h-old neonate larvae), and plant substrate (Red Delicious apple leaves), the sources of the difference in spinetoram field performance between both studies were due to differences in the field conditions, application conditions, levels of resistance, and time of exposure (120 h in the current study and 168 h in the Sial and Brunner 2010b study).

In contrast, spinetoram showed a low field performance by controlling *Drosophila suzukii* and apple maggot for only the first 7-day post-application when the flies were exposed to treated blueberry and apple fruit, respectively [20,26]. The same compound showed a poor field performance when it failed to control *Drosophila suzukii* even for the first 7-day post-application when the flies were exposed to treated strawberry fruit [20].

Based on the high field performance of spinetoram that was documented in the current study, this insecticide appears to be an excellent chemical tool to control *C. rosaceana* field populations in Michigan apple and cherry orchards. However, resistance development against this insecticide should be monitored periodically for further increases in the resistance level. This can be accomplished once annually, if possible, by collecting *C. rosaceana* field populations from the targeted orchards and then determining the resistance levels in them against spinetoram. In the spinetoram application, a 14-day post-application should be considered for the re-application interval for this compound.

### 4.4. Chlorantraniliprole

Chlorantraniliprole is a broad-spectrum insecticide with activity in a wide range of pests in many crops. It has very low water solubility, a low vapor pressure, and is stable to photolysis (except in water) [43]. Chlorantraniliprole is highly rainfast plus it has translaminar activity in which both surface and inner residues are relatively protected, allowing it to provide long-lasting crop protection [34,36].

The long-lasting leaf residues documented in the current study over all post-application intervals is consistent with the findings reported by [34] and [44]. The absence of significant differences in larval mortality between susceptible and apple *C. rosaceana* populations at all post-application intervals (except 21 days) was expected to be due to the high efficacy and long-lasting residues of chlorantraniliprole overcoming the documented 4.7-fold resistance of the apple field *C. rosaceana* population in the baseline study (Figure 1) over nearly three weeks until 67.1% of the 1-day post-application leaf residues had been degraded. This means the presence of nearly ≥40% of the chlorantraniliprole field rate residue is enough to control the *C. rosaceana* field populations effectively.

Poor field performance of chlorantraniliprole was previously documented when this compound failed to reduce the apple maggot larval emergence significantly, compared to the untreated control, in the chlorantraniliprole-treated apple fruit, even at 1-day post-application [38].

In contrast, a higher chlorantraniliprole field performance than that recorded in the current study was documented by SialandBrunner [8], who reported that the chlorantraniliprole-treated apple foliage caused 100% mortality in *C. rosaceana* neonate larvae (˂48 h old) over 38-day post-application. Since SialandBrunner [8] and the current studies had tested the same compound (chlorantraniliprole), species (*C. rosaceana*), life-stage (˂48 h-old neonate larvae), time of exposure (168 h), and plant substrate (Red Delicious apple leaves), the source of the difference in chlorantraniliprole field performance between both studies is likely due to the differences in the levels of resistance in the tested populations, field conditions, and application conditions. Where, for example, SialandBrunner [8] had tested a laboratory-susceptible population of *C. rosaceana* with no level of resistance, the current study had tested in addition to the susceptible population a field population with a resistance ratio of 4.7-fold compared to the susceptible population.

The moderate lethality in the current study should not be considered as a final conclusion of the ability of chlorantraniliprole to provide fruit protection. The current study evaluated chlorantraniliprole only based on the lethal activity; however, the compound has lethal and sublethal effects, e.g., delaying oviposition [45]. Based on the results of the current study, 14-day post-application should be considered for the re-application interval for this compound.

### 4.5. Indoxacarb

Indoxacarb is a broad-spectrum and effective insecticide against a wide range of pests, especially lepidopteran larvae, in many crops [46]. It is a non-volatile compound with a low vapor pressure, low water solubility, and a complex degradation profile (except in soil where it has a rapid degradation rate) [47]. Indoxacarb is also moderately susceptible to wash-off from precipitation but its capability of plant cuticle penetration helps to reserve a portion of its active ingredient in the plant leaves and fruit cuticle [36].

The failure of indoxacarb (practical resistance) to control *C. rosaceana* in the field, especially in the apple trial, was likely due to the high resistance levels in the tested *C. rosaceana* populations uncovered in the baseline study [1] (Figure 1), which was also found to be a complex resistance case with the involvement of three metabolic resistance mechanisms [4], despite the flat degradation in leaf residues over time. Wise et al. [17] also found that indoxacarb residues remained stable over time (up to two weeks) in a sufficient quantity to cause lethal effects.

Unlike the poor field performance in the current study, indoxacarb showed good field efficacy with 14-day post-application control of *Forficula auricularia* (European earwig) when the adults were exposed to treated apple foliage [48]. Similarly, indoxacarb showed a good field performance with control of plum curculio over 14-day post-application when the adults were exposed to treated apple fruit [17]. A previous trial in tart cherry was similar to the current cherry trial where indoxacarb showed a low field performance and provided fruit protection for only 7-day post-application against plum curculio when the adults were exposed to treated cherry fruit [39].

The differences in the field performance of indoxacarb that were documented in different studies, including the current study, is assumed to be due to the differences in the field conditions, application conditions, tested species, tested life-stage, levels of resistance, and plant substrate.

Even though indoxacarb is not labeled for use against *C. rosaceana* in fruit orchards, growers should take this indoxacarb field failure into account when using this compound in the control programs of other pests, e.g., plum curculio, and not expect incidental control of resident leafrollers.

### 4.6. Emamectin Benzoate

The emamectin benzoate insecticide is a derivative of abamectin, which is effective against numerous pests. It is a semi-synthetic compound that is isolated from the fermentation of the soil actinomycete *Streptomyces avermitilis* [49,50]. Emamectin benzoate has a very low solubility in water and a low vapor pressure, and is sensitive to photodegradation [49,50]. Despite the photolysis sensitivity, emamectin benzoate has translaminar activity in which it is able to penetrate the leaf cuticle and move into and across leaf tissues, which means the compound’s active ingredient is protected from photolysis inside the leaf tissues [50].

The low field performance in the cherry and apple trials were not only due to the documented resistance levels in the *C. rosaceana* field populations against this compound in the baseline study (Figure 1), but also due to the short residuality of this compound as a foliar application. The rapid decline in surface residues under the field conditions was expected since emamectin benzoate is highly sensitive to photolysis. However, the lack of detectable residues at the 7-day sample and after may in part have been a result of the residue sample preparation methods used in the current study, where some portions of sub-cuticular residues from internal leaf tissues may have remained unmeasured. It is interesting to note that when alternative delivery systems, such as the trunk injection, are used to apply emamectin benzoate, *C. rosaceana* can be effectively controlled over two growing seasons [28,51]. This highlights the sensitivity of this compound to environmental degradation on the tree canopy.

Higher emamectin benzoate field performance, compared to its field performance in the current study, was documented by SialandBrunner [7] for *C. rosaceana* and for codling moth and oriental fruit moth [52] when neonate larvae were exposed to treated apple foliage or fruit. SialandBrunner [19] found that emamectin benzoate-treated apple foliage caused 100% mortality in the *C. rosaceana* neonate larvae (˂48 h old) up to 10-day post-application. Since the current and SialandBrunner [19] studies had tested the same compound (emamectin benzoate), same species (*C. rosaceana*), same life-stage (˂48 h-old neonate larvae), and same plant substrate (Red Delicious apple leaves, in the case of current apple trial), the sources of the difference in emamectin benzoate field performance between both studies are expected to be due to the tested populations.

Emamectin benzoate is registered as a foliar spray in the U.S. against numerous pests in apples [53]. Based on the results of the current study, 7-day post-application should be considered as the recommended re-application interval for this compound if there is a high pest infestation that needs a supplementary insecticide application.

## 5. Conclusions

Compounds demonstrating low levels of field-evolved resistance in *C. rosaceana* populations from apples and cherries orchards did not result in practical resistance in the field-based trial (i.e., a lack of control under field conditions). However, compounds with high levels of resistance of *C. rosaceana* resulted in practical resistance in both resistant populations. Only chlorantraniliprole and indoxacarb showed long-lasting residues with measurable leaf residues over all post-application intervals while the leaf residues of the other compounds had largely degraded within the first 7 days.

## Figures and Tables

**Table 1:** The details of the compounds that were tested in apple and cherry field trials.

			Treatment	
Trial	Trade Name	Active Ingredient	AI/Acre (lb)	Company
Apple and Cherry	Imidan 70W	Phosmet	3	Gowan Company, Yuma, AZ, USA
Apple and Cherry	Bifenture 10DF	Bifenthrin	1	United Phosphorus, Inc., King of Prussia, PA, USA
Apple and Cherry	Delegate 25WG	Spinetoram	0.375	Dow AgroSciences, Indianapolis, IN, USA
Apple	Altacor 35WG	Chlorantraniliprole	0.281	I.E. du Pont De Nemours and Co., Wilmington, DE, USA
Apple and Cherry	Avaunt 30WG	Indoxacarb	0.375	I.E. du Pont De Nemours and Co., Wilmington, DE, USA
Apple and Cherry	Proclaim 5SG	Emamectin benzoate	0.3	Syngenta Crop Protection Inc., Greensboro, NC, USA

100 Gallons per acre (GPA).

**Table 2:** LOD and LOQ values for each treatment compound.

Chemical	LOD (µg/g)	LOQ (µg/g)
Phosmet	0.001	0.005
Bifenthrin	0.005	0.016
Spinetoram	0.121	0.400
Chlorantraniliprole	0.015	0.050
Indoxacarb	0.001	0.002
Emamectin benzoate	0.010	0.050

LOD = Limit of detecting a peak. LOQ = Limit of quantifying a peak.

## Data Availability

The data presented in this study are available from the corresponding author on a reasonable request.
